# Synthetic Strigolactone GR24 Improves *Arabidopsis* Somatic Embryogenesis through Changes in Auxin Responses

**DOI:** 10.3390/plants10122720

**Published:** 2021-12-10

**Authors:** Mohamed Elhiti, Mohammed M. Mira, Kenny K. Y. So, Claudio Stasolla, Kim H. Hebelstrup

**Affiliations:** 1 Department of Agroecology, University of Aarhus, Forsøgsvej 1, 4200 Slagelse, Denmark; melhiti@farmersbusinessnetwork.com; 2 Department of Botany, Faculty of Science, Tanta University, Tanta 31527, Egypt; mohammed.mira@umanitoba.ca; 3 Department of Plant Science, University of Manitoba, Winnipeg, MB R3T 2N2, Canada; sok@myumanitoba.ca

**Keywords:** quantitative PCR, 2,4-D, TIS108, *MAX3*, *MAX4*, *ARFs*, *WUS*, *SERK*

## Abstract

Somatic embryogenesis in *Arabidopsis* encompasses an induction phase requiring auxin as the inductive signal to promote cellular dedifferentiation and formation of the embryogenic tissue, and a developmental phase favoring the maturation of the embryos. Strigolactones (SLs) have been categorized as a novel group of plant hormones based on their ability to affect physiological phenomena in plants. The study analyzed the effects of synthetic strigolactone GR24, applied during the induction phase, on auxin response and formation of somatic embryos. The expression level of two SL biosynthetic genes, *MORE* *AXILLARY GROWTH 3* and *4 (MAX3* and *MAX4*), which are responsible for the conversion of carotene to carotenal, increased during the induction phase of embryogenesis. *Arabidopsis* mutant studies indicated that the somatic embryo number was inhibited in *max3* and *max4* mutants, and this effect was reversed by applications of GR24, a synthetic strigolactone, and exacerbated by TIS108, a SL biosynthetic inhibitor. The transcriptional studies revealed that the regulation of GR24 and TIS108 on somatic embryogenesis correlated with changes in expression of *AUXIN RESPONSIVE FACTORs 5, 8, 10, and 16,* known to be required for the production of the embryogenic tissue, as well as the expression of *WUSCHEL* (*WUS*) and *Somatic Embryogenesis Receptor-like Kinase 1* (*SERK1*), which are markers of cell dedifferentiation and embryogenic tissue formation. Collectively, this work demonstrated the novel role of SL in enhancing the embryogenic process in *Arabidopsis* and its requirement for inducing the expression of genes related to auxin signaling and production of embryogenic tissue.

## 1. Introduction

Strigolactones (SLs) are semiochemicals first identified as stimulants of seed germination in species of the parasitic weed *Striga* [1], and later as potent branching factors for mycorrhizal fungi [2,3]. Recently, SLs have been categorized as a novel group of plant hormones based on their ability to inhibit lateral shoot branching [4,5]. They have potential roles as regulators of plant development by influencing root architecture [6,7], leaf senescence [8], and secondary growth [9]. They are also known to promote germination in thermo-inhibited seeds, break secondary dormancy in *Arabidopsis* seeds [10], and alter photomorphogenic development [11]. Widely distributed and ubiquitous among plant species ranging from liverworts to flowering plants [12], SLs are mainly synthesized in root cells and translocated acropetally to all tissues and organs [13]. Characterization of *Arabidopsis* mutants with excess branching, such as *more axillary growth* (*max1*, *max2*, *max3*, and *max4*), contributed to the identification of enzymes in the SLs MAX-biosynthetic pathway. Cloning and sequencing studies further revealed that *MAX3* and *MAX4* encode carotenoid cleavage dioxygenases (CCD7 and CCD8, respectively), while *MAX1* encodes a cytochrome P450 [14]. Biochemical studies showed that these mutants are deficient in SL and exogenous application of GR24, a synthetic analogue to SL, and rescued the excess branching phenotype of *max3* and *max4,* but not that of *max2* [15]. This observation suggested that *MAX3* and *MAX4* are components of the SL biosynthetic pathway, while *MAX2* is more likely a component of the SL signal-transduction pathway. Another characterized SL biosynthetic gene is *MAX1*, which catalyzes the conversion of carlactone to SL, as evidenced by the accumulation of the latter in *max1* plants [14].

Embryogenesis (sexual plant reproduction) is a biological process in which double fertilization forms a single-celled zygote and a founder cell of the endosperm [16]. Subsequently, the zygote develops into a zygotic embryo. If this process is initiated from somatic cells, it is referred to as somatic embryogenesis (or in vitro-induced embryos). Somatic embryos are structurally similar to their zygotic counterparts, but unlike zygotic embryos, which are embedded in the material tissue, they are exposed. Due to this characteristic, somatic embryogenesis is considered an efficient and reliable tool to study embryogenesis at the molecular, biochemical, and cellular levels [16,17,18]. As reviewed by Elhiti et al. (2013), the somatic embryogenic process is initiated by the acquisition of totipotency followed by dedifferentiation and development of an “embryogenic commitment” facilitated by the action of signal molecules [16], and specifically plant growth regulators [19]. In more than 80% of cases, auxin is utilized as the inductive signal required for the dedifferentiation step and formation of the embryogenic tissue from the cultured explants [20]. Key genetic components of the auxin-signaling pathway are the *AUXIN RESPONSE FACTORs* (ARFs) [21]. Wójcikowska and Gaj (2017) suggested that differential regulation of ARFs during the induction phase of somatic embryogenesis is crucial for the successful formation of embryos [19], as evidenced by the induction of *ARF5*, *ARF6*, *ARF8*, *ARF10*, *ARF16*, and *ARF17,* and the concomitant suppression of *ARF1*, *ARF2*, *ARF3*, *ARF11*, and *ARF18*. Involvement of ARFs in developmental processes is not restricted to embryogenesis, as they contribute to the development of several postembryonic events, including flowering, leaf senescence, root development, vascular tissue formation, and abaxial identity of organs [22].

The regulation of plant development by SLs is often linked to the action of plant growth regulators, including auxin [6,23,24]. Production of SLs during apical dominance is induced by auxin [25,26], while applications of the synthetic SL, GR24, regulates the expression of auxin-related genes in young *Arabidopsis* seedlings [27]. These observations suggest that SLs might interfere with auxin signaling during somatic embryogenesis and ultimately influence the formation of somatic embryos. To test this hypothesis, we examined the in vitro embryogenic response of *Arabidopsis* mutants deficient in SLs, and the effects of pharmacological treatments altering the level of SLs during the induction phase of somatic embryogenesis. In addition, the regulation effects of GR24 (a synthetic strigolactone) and TIS108 (a SL biosynthetic inhibitor) in expression of *AUXIN RESPONSIVE FACTORs 5, 8, 10,* and *16,* as well as somatic embryogenesis marker genes *WUSCHEL* (*WUS*) and *Somatic Embryogenesis Receptor-like Kinase 1* (*SERK1*), were investigated. Moreover, the responsiveness of explants with or without SL to auxin was validated.

## 2. Materials and Methods

### 2.1. Plant Material and Treatments

**Figure 1:**
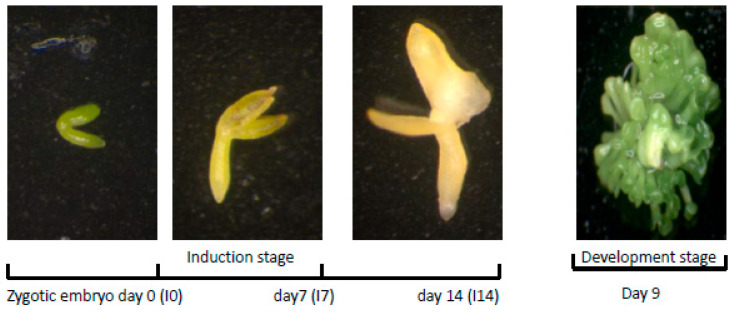
Schematic representation of the somatic embryogenic process in *Arabidopsis*. Immature zygotic embryos were cultured for 14 days on a 2,4-D-containing induction medium required for the formation of embryogenic tissue by immature embryos. Continuation of embryo development was achieved by transferring the explants on development medium devoid of 2,4-D. Fully developed somatic embryos were visible after 9 days.

*Arabidopsis* ecotype (Col–0) seeds, including *max1*, *max3–9,* and *max4* mutants, were obtained from the Nottingham *Arabidopsis* Stock Centre (NASC). *Arabidopsis* seeds were surface-sterilized (20% commercial bleach solution with a drop of Tween20, 10 min, followed by three washes of distilled water) and plated on germination medium (half-strength MS, pH 5.8 and supplemented by 6% agar, per Murashige and Skoog, 1962). The Petri dishes (100 mm × 20 mm, Sigma Millipore) were kept at 4 °C in darkness for 2 days to overcome dormancy, and then transferred to a growth room maintained at 20–22 °C with a 16 h light/8 h dark cycle (cool fluorescent light, 40 μmol m^−2^ s^−1^) for 7 days. Young seedlings were transplanted into vermiculite and grown in a growth chamber under the same conditions as described above until harvesting of immature siliques. Somatic embryo induction was performed using a method based on that described by Bassuner et al., 2007 [28]. The collected siliques were surface sterilized by immersing them in 70% ethanol for 30 s and washing 2× with sterilized water, and then soaking in a 50 mL Falcon tube containing 45 mL of 10% commercial bleach on a rotary shaker at 100 RPM. After 10 min, the siliques were washed 5× using distilled sterilized water. Then, the siliques were collected by sterilized forceps in a Petri dish (50 × 14 × 54 mm) and kept overnight in the 4 °C fridge. Immature zygotic embryos were dissected using simple microscopy under aseptic conditions. The bent-cotyledon (torpedo stage) zygotic embryo (Figure 1) was used for explants; 15 explants were incubated on induction media. After 14 days, the explants were transferred to development medium and fully mature somatic embryos were counted after 9 days on development medium [29]. The optimal concentration of GR24 was selected based on the highest number of developed embryos. However, the TIS108 was selected at the highest concentration at which the minimum number of embryos were developed. Concentrations for GR24 (10 nM) and TIS108 (50 nM), an SL analog and an SL biosynthetic inhibitor, were empirically determined. Both compounds were dissolved in 3–5 drops of acetone and volume adjusted with water. Solutions (10 μL) were directly dispensed every other day on the explants during the 14 days of the induction process according to the treatment simultaneously. Acetone (diluted with water) was used for control treatment. Each experiment was performed using three biological replicates, each consisting of 15 explants.

For *Arabidopsis* somatic embryogenesis, the induction medium was LV macronutrients with MS micronutrients supplemented with full-strength B5 vitamins, 4.5 µM 2,4-D (2,4-dichlorophenoxyacetic acid, Sigma Millipore), 20 g/L sucrose, and 3 g/L phytagel; the pH was adjusted to 5.8 by 0.1 M NaOH. The development media preparation was B5 medium fortified with 30 g/L sucrose, and the pH was adjusted to 5.8. Then, 3 g/L phytagel was added. *Arabidopsis* seed germination medium was half-strength of the full MS supported by 10 g/L sucrose and 6 g/L agarose, and the pH values were adjusted to 5.8 (as described previously). After media preparation, all media were autoclaved and poured into Petri dishes (100 mm × 20 mm, Sigma Millipore). The dishes were left open for about 30 min to prevent condensation. All salts and vitamins were ordered from Plantmedia, Bioworld.

### 2.2. RNA Isolation and Gene Expression Analysis

A Spectrum Plant Total RNA kit (Sigma-Aldrich, St. Louis, MO, USA) was used to isolate total RNA from the initial explants at days 0, 7, and 14 during the induction, and at day 3 and 9 during development stages. The concentration and purity of RNA were evaluated with an ND-1000 spectrophotometer (NanoDrop, ThermoFisher). To prevent DNA contamination, an On-Column DNase I Digestion Set (Sigma-Aldrich) was employed to remove trace amounts of DNA, following the manufacturer’s instructions. First-strand cDNA was produced in a 20 µL reaction volume with 1 µg of RNA using a Maxima First Strand cDNA Synthesis Kit (ThermoFisher, Waltham, MA, USA).

Real-time quantitative RT-PCR (qRT-PCR) was used to measure the levels of transcripts. The product of the reverse transcription was diluted using a 3:1 ratio (water:cDNA), then 2 µL was used for each reaction. The qRT-PCR was carried out in a 10 µL reaction volume using a LightCycler^®^ 480 SYBR™ Green I Master (Roche) kit, a LightCycler^®^ 480 Multiwell Plate 96, and Multiwell Sealing Foil (Roche).

A LightCycler^®^ 480 System (Roche) real-time detection system was used with the following reaction conditions: denaturation one repeat of 5 min at 95 °C, followed by 40 repeats of 10 s at 95 °C, 20 s at 58 °C, and 10 s at 72 °C. Denaturation for melt curve analysis was conducted at 95 °C, followed by 5 s at 65 °C for 1 min, and 98 °C (0.11 °C/s for fluorescence measurement). Cooling was at 40 °C for 10 s. Relative transcript levels were calculated and normalized using the ELONGATION FACTOR1α (AT1G07920) as the internal control [30]. Fold-change values were calculated using the comparative 2^−ΔΔCt^ method described by Livak and Schmittgen, 2001 [31]. The primers (Supplementary Table S1) were designed using the Primer Quest™ Tool (https://www.idtdna.com/pages/tools/primerquest).

### 2.3. Auxin Sensitivity Assay

Immature zygotic explants were cultured on induction medium containing different levels of 2,4-D, ranging from 0 to 9 µM, for 14 days. After transfer onto development medium, somatic embryos were counted after 9 days. The embryogenic responses of WT, *max3–9*, and *max4* explants were also calculated as a function of the number of somatic embryos produced by each explant against the concentration of 2,4 D applied in the induction medium. For GR24 and TIS108 application to explants, 10 μL solutions with the proper concentration were directly dispensed every other day on the explants simultaneously during the 14 days of the induction process according to the treatment.

### 2.4. Statistical Analysis

The Tukey’s post hoc test for multiple variances was used to calculate any significant differences (at *p* = *0.05*) between treatment combinations compared in these studies [32].

## 3. Results

### 3.1. Somatic Embryogenesis Is Influenced by the Expression of MAX3–9 and MAX4

Somatic embryogenesis in *Arabidopsis* was a two-step process with an induction and developmental phase. In the 14-day induction phase, the bent-cotyledonary stage zygotic embryos were placed on a medium containing 2,4-D, required for the formation of proembryogenic masses generated from the abaxial sides of the cotyledons. Continuation of the embryogenic process was achieved by transferring the explants onto development medium devoid of plant growth regulators; fully mature somatic embryos were visible after 9 days (Figure 1). The requirement for SL during the embryogenic process was examined using explants from genotypes with attenuated expression of genes encoding known enzymes of the SL biosynthetic pathway *MAX1,* which encodes a cytochrome P450, as well as *MAX3–9* and *MAX4,* which encode the carotenoid cleavage dioxygenases 7 and 8, respectively [14]. All three mutants exhibited a significant reduction in SL content [14].

**Figure 2:**
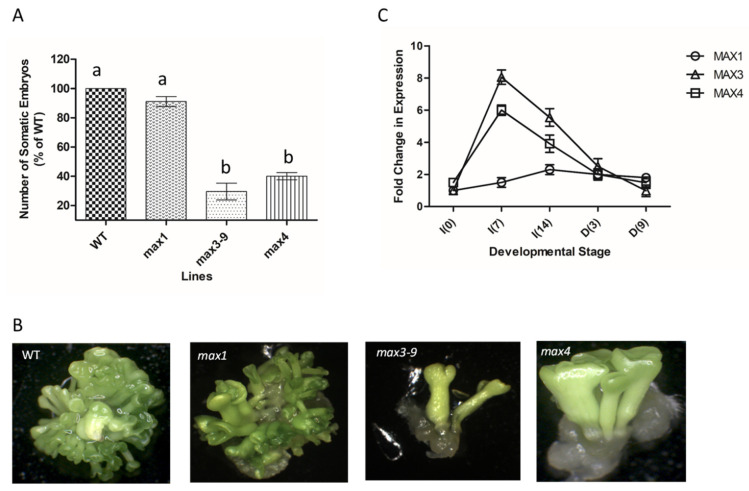
(**A**) Number of somatic embryos produced by WT tissue and *max1*, *3–9*, and *4* tissues. Values are means ± SE of three biological replicates (*n* = 15). Letters on bars indicate statistically significant differences (*p* < 0.05). (**B**) Micrographs showing the number of fully developed somatic embryos from tissue in (**A**). (**C**) The fold change in expression level of *MAX1*, *3–9*, and 4 at different time points during the induction (I) and developmental (D) stages of embryogenesis. Values are means ± SE of three biological replicates (*n* = 15).

Loss of function of the *MAX1* gene did not show significant effects on the number of somatic embryos produced. However, loss of function of *MAX3–9* and *MAX4* genes inhibited the somatic embryogenesis process (Figure 2A,B). In both genotypes, somatic embryo production was reduced by more than half compared to that recorded in the WT. The observed changes in embryogenic competence as a result of these mutations prompted the analyses of transcript levels of the three genes at different times during the induction and developmental phases of embryogenesis. The transcript levels of *MAX3–9* and *MAX4* increased significantly during the first 7 days of the induction phase before declining during the subsequent days of induction and development (Figure 2C). The expression level of *MAX1* did not show marked changes, with a very small increase between days 7 and 14 of induction.

Collectively, these results indicated that expression of *MAX3–9* and *MAX4* is required for *Arabidopsis* somatic embryogenesis, and their increased expression during the first days of induction suggested their involvement during the initial stages of embryogenic tissue formation.

### 3.2. Strigolactones (SLs) Are Required for the Formation of Somatic Embryo

**Figure 3:**
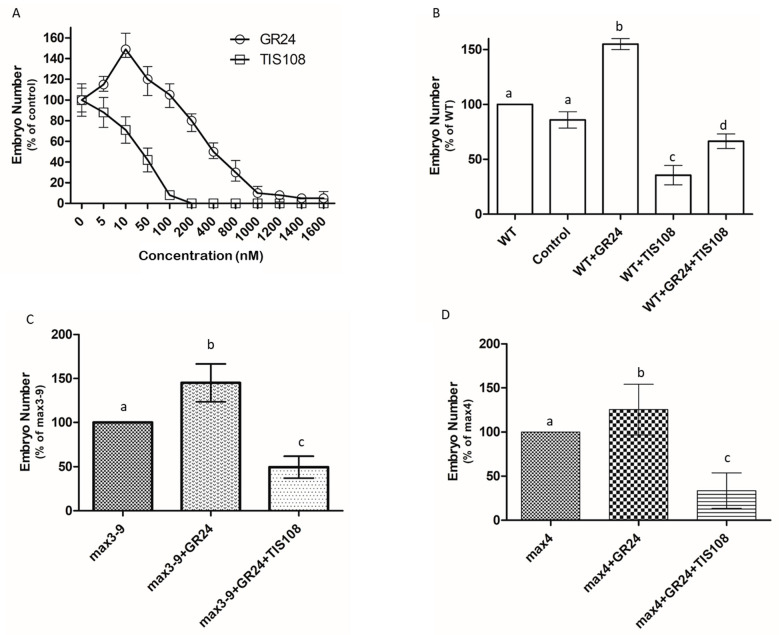
Requirement of SL for somatic embryogenesis in *Arabidopsis*. (**A**) Effects of different concentrations of GR24 and TIS108 applied in the induction medium on the number of somatic embryos. Values are means ± SE of three biological replicates (*n* = 15). (**B**) Effects of GR24 (50 nM) and/or TIS108 (10 nM) on the number of somatic embryos produced by the WT tissue. A mock experiment was conducted using water (Control). Values are means ± SE of three biological replicates (*n* = 15). Letters on bars indicate statistically significant differences (*p* < 0.05). (**C**) Effects of GR24 (50 nM) or TIS108 (10 nM) on the number of somatic embryos produced by *max3–9* tissue. Values are means ± SE of three biological replicates (*n* = 15). Letters on bars indicate statistically significant differences (*p* < 0.05). (**D**) Effects of GR24 (50 nM) or TIS108 (10 nM) on the number of somatic embryos produced by *max4* tissue. Values are means ± SE of three biological replicates (*n* = 15). Letters on bars indicate statistically significant differences (*p* < 0.05).

To further confirm the requirement of SLs in the somatic embryogenic process, we examined the effects of exogenous applications of GR24, a synthetic SL analog, and TIS108, a SL biosynthetic inhibitor, during the induction phase. An initial dose–response curve was generated in WT tissue to evaluate the optimal concentration of both compounds. Applications of GR4 in the range of 5–50 nM increased the number of somatic embryos, while higher concentrations diminished their number (Figure 3A). A dose-dependent inhibition of TIS108 on the number of embryos was observed between 5–100 nM. No embryos were produced when TIS108 was applied at concentrations higher than 200 nM.

To evaluate whether the TIS108 repression of embryogenesis was mainly due to its inhibitory effect on SL biosynthesis, we performed co-applications of GR4 and TIS108. We first confirmed that the solvent (acetone) used to dissolve the two chemicals (control) had no effects on the number of somatic embryos produced by WT tissue (Figure 3B). While GR4 (50 nM) increased the number of somatic embryos, TIS108 (10 nM) diminished embryo production, and this effect was partially reverted by the co-application of GR24.

Application of GR4 was also able to augment the number of embryos in the *max3–9* and *max4* explants, and the inclusion of TIS108 suppressed the production of embryos well below that of the untreated explants (Figure 3C,D).

Taken together, these results suggested that SL is required for the generation of somatic embryos, with GR24 elevating the number of embryos and TIS108 suppressing the ability of the explants to generate embryos. These effects were also confirmed in the *max3–9* and *max4* mutants, characterized by reduced levels of endogenous SL.

### 3.3. Strigolactones Affect Auxin Response during the Induction Phase Enhancing Somatic Embryogenesis

**Figure 4:**
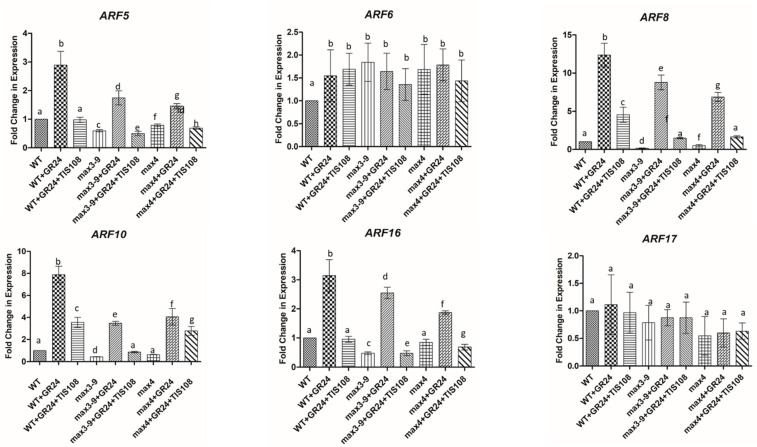
Fold change in expression level of *ARF5, 6, 8, 10, 16,* and *17* in WT, *max3–9*, and *max4* tissue after 7 days of induction. Tissue was also treated with GR24 (50 nM) and/or TIS108 (10 nM). Values are means ± SE of three biological replicates (*n* = 15). Letters on bars indicate statistically significant differences (*p* < 0.05).

Auxin is a key regulator of somatic embryogenesis; it induces cell dedifferentiation and the formation of embryogenic tissue during the induction phase [33]. These effects are mediated by AUXINRESPONSE FACTORs (ARFs) regulating SKP/CULLIN/F-BOX-ubiquitin (SCF^TIR1^) [19]. To examine the link between SL and auxin response, the transcript levels of specific ARFs, known to vary in expression during embryogenesis [34], were measured on day 7 in the induction medium following treatments with GR24 and TIS108. This day was chosen based on the peak in *MAX3–9* and *4* expression levels (Figure 2B) and the auxin-mediated formation of the embryogenic tissue, which was apparent at day 7 [30]. The transcript levels of several ARFs: *ARF5*, *ARF8*, *ARF10*, and *ARF16* genes, were upregulated by GR24 in WT, *max3–9*, and *max4* explants, and these effects were partially reversed when TIS108 was coapplied (Figure 4). No major fluctuations in transcript levels were observed for ARF6 and ARF17.

**Figure 5:**
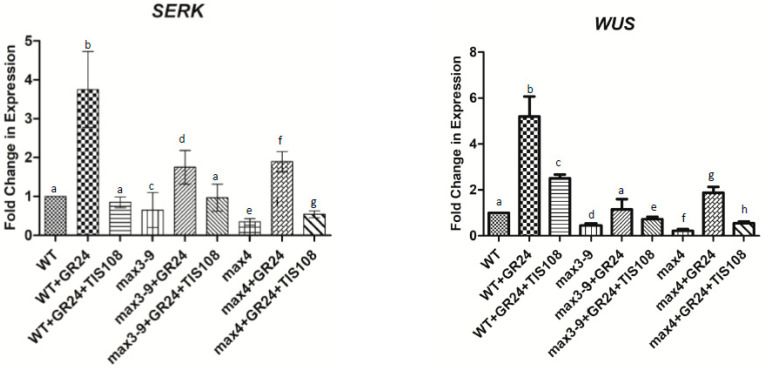
Fold change in expression levels of *SERK1* and *WUS* in WT, *max3–9*, and *max4* tissue after 7 days of induction. Tissue was also treated with GR24 (50 nM) and/or TIS108 (10 nM). Values are means ± SE of three biological replicates (*n* = 15). Letters on bars indicate statistically significant differences (*p* < 0.05).

**Figure 6:**
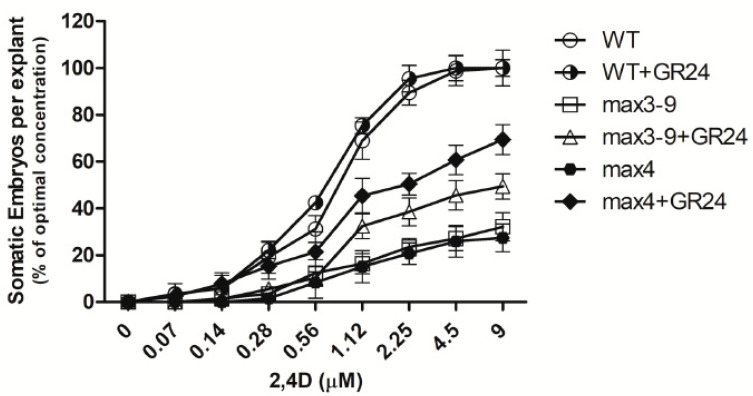
Effects of different concentration of 2,4-D on the number of somatic embryos generated from WT, *max3–9*, and *max4* explants. Tissue was also treated with GR24 (50 nM). Values are means ± SE of three biological replicates (*n* = 15). Letters on bars indicate statistically significant differences (*p* < 0.05).

To further assess whether the GR4 and TIS108 regulation of *ARF5, 8, 10,* and *16* was linked to the acquisition of embryogenic competence, we also measured the expression of *WUSCHEL* (*WUS)* and *Somatic Embryogenesis Receptor-like Kinase 1* (*SERK1*), two well characterized markers of embryogenic competence downstream of auxin signaling [35]. The transcript levels of both *WUS* and *SERK1* exhibited a very similar pattern to that described for the four ARFs (Figure 5), thus suggesting the requirement for SL for the activation of auxin responses and embryogenic competence. To further confirm this notion, we also assessed whether applications of GR4 to *max3–9* and *max4* explants would increase auxin sensitivity by lowering the requirement of auxin needed to generate somatic embryos. The required level of auxin needed to produce somatic embryos was significantly reduced when GR4 was included in the induction medium of both *max3–9* and *max4* explants and the wild type (Figure 6).

## 4. Discussion

The developmental plasticity of plant somatic cells allows them to dedifferentiate and embark on new developmental pathways, resulting in the production of tissues, organs, or new plants. This inherent characteristic is best exemplified in tissue culture, where somatic cells can be induced to produce embryos through a process known as somatic embryogenesis. In this process, the auxin 2,4-D acts as the inductive signal promoting dedifferentiation of somatic cells and formation of embryogenic tissue occurring in the induction medium [36,37]. This notion was also confirmed by the numerous auxin-related genes induced during the initial phases of somatic embryogenesis in a variety of systems, including *Picea glauca.* (Moench) Voss [38], *Zea mays* L. [39], *Glycine max* (L.) Merr. [40], *Solanum tuberosum* L. [41], and *Arabidopsis thaliana* [42].

The formation of embryos in culture is influenced by environmental stresses [29,43], and SLs participate in adaptive plant growth responses to environmental conditions [44,45]. As some of these responses are integrated with auxin signaling, it was the purpose of this work to examine the involvement of SLs in somatic embryogenesis and their effect on auxin responses required for the development of somatic embryos. Several pieces of evidence indicated that SL is required for somatic embryogenesis. First, the number of somatic embryos was significantly diminished in *max3–9* and *max4* tissues relative to their WT counterpart. MAX3–9 an MAX4 are well-characterized SL biosynthetic genes that encode the carotenoid cleavage dioxygenases 7 and 8, respectively [14,46,47]. The observation that another SL biosynthetic gene, *MAX1,* which encodes a cytochrome P450 [14], did not seem to influence embryogenesis when knocked down was interesting. It is possible that MAX1 catalyzes the conversion of carlactone to carlactonic acid [47]. A mutation of this enzyme would cause the accumulation of carlactone, which has been shown to retain a biological activity comparable to that of SL [46], thus having similar effects on somatic embryogenesis. Additional evidence for the SL requirement during somatic embryogenesis comes from the increased expression of *MAX3–9* and *MAX4* at day 7 of the induction phase, corresponding to the auxin-mediated emergence of the embryogenic tissue [30], and the effects of pharmacological treatments. Applications of the SL analog GR24 increased the number of embryos, and this effect was significantly reduced by the coapplication of the SL inhibitor TIS108; this regulation was observed in WT tissue, as well as in *max3–9* and *max4* tissue. The SL-like activity of GR24 was demonstrated by Wu et al. (2017), who were able to augment the number of somatic embryos from leaf explants deficient in SL [47]. TIS108 is a triazole-type SL biosynthesis inhibitor that suppresses the level of 2’-epi-5-deoxystrigol (epi-5DS) in rice [48] and is routinely used in studies aimed at suppressing SL level [49,50].

The effects of SL alterations by GR24 and TIS108 on somatic embryogenesis seems to be linked to the expression of several ARFs participating in auxin signaling. Characterized as key signals present in clusters of cells at the base of the cotyledons in the explants, ARFs regulate the formation of proembryonic masses, and ultimately the production of somatic embryos [19]. These effects were ascribed to changes in auxin responses. ARFs contain a B3-type DNA-binding domain that binds to the TCTCTC motif (Aux RE) found in the promoters of auxin-responsive genes [22,36]. The mechanism of auxin-induced gene activation has been well characterized. In the absence of auxin, the Aux/IAA protein interacts with its partner ARF, thereby halting any ARF activity; while in the presence of auxin, the Aux/IAA protein is degraded through ubiquitination by the SKP-Cullin-F-boxTIR1/AFB (SCFTIR1/AFBs) E3 ubiquitin ligase complex, which contains the auxin receptor TRANSPORT INHIBITOR RESPONSE1(TIR1)/AUXIN RECEPTOR F-BOX PROTEINS (AFBs) [51]. Our work focused specifically on ARF5, 6, 8, 15, 16, and 17, which are known to be induced during the induction phase of somatic embryogenesis [19]. Here, we show that the expression of ARF5, 8, 10, and 16 were regulated by SL, with GR4 increasing their expression, while TIPS108 acted as a suppressor. Based on the ARFs’ mode of action described above, it is plausible to speculate that the GR24 induction of ARF5, 8, 10, and 16 might confer auxin hypersensitivity. This notion was supported by the increased sensitivity to auxin exhibited by *max3–9* and *max4* tissue following applications of GR24.

In agreement with the SL regulation of auxin response, as well as the requirement for auxin in the formation of embryogenic tissue, here we showed that SL is needed for the expression of *WUS* and *SERK1*, two well-characterized molecular markers linked to the production of embryogenic tissue [52]. Initially characterized in relation to the function of the shoot apical meristem [53], *WUS* is expressed in those regions of the explants forming embryogenic tissue [54]. Its expression is induced by auxin, and its over-expression is sufficient to increase the production of somatic embryos [55]. Another key marker conferring embryonic competence is SERK. Initially characterized in carrot suspension cultures, where it was specifically expressed in cells developing into somatic embryos [56], *SERK1* was later described in many embryogenic systems, including *Dactylis glomerata* L. [57], *Arabidopsis thaliana* [58], *Medicago truncatula* Gaertn. [59], *Helianthus annuus* L. [60], *Ocotea catharinensis* Mez. [61], *Citrus unshiu* Marcow [62], and *Tilia amurensis* [63]. In all these systems, *SERK1* expression was linked to the formation of embryogenic tissue and embryogenic competence of the explants.

In conclusion, this study demonstrated the requirement of SL for somatic embryogenesis in *Arabidopsis*. The increased number of somatic embryos observed following applications of GR24 correlated with the induction of several ARFs that modulated auxin responses, and molecular markers required for the formation of embryogenic tissue. In addition to documenting a novel link between SL and auxin in embryogenesis, this study provided a simple protocol for increasing the number of embryos that could be useful for propagation of species recalcitrant to tissue culture. We believe that these findings may be an important stepping stone in the future direction of understanding the genetic control of SL and auxin biosynthesis, as well as improving induction of totipotency for plant-transformation technologies.

## Data Availability

Data sharing not applicable.

## Supplementary Materials

The following are available online at https://www.mdpi.com/article/10.3390/plants10122720/s1, Table S1: List of primers used in this study.
